# Induction of eosinophil apoptosis by hydrogen peroxide promotes the resolution of allergic inflammation

**DOI:** 10.1038/cddis.2014.580

**Published:** 2015-02-12

**Authors:** A C Reis, A L Alessandri, R M Athayde, D A Perez, J P Vago, T V Ávila, T P T Ferreira, A CS de Arantes, D de Sá Coutinho, M A Rachid, L P Sousa, M A Martins, G B Menezes, A G Rossi, M M Teixeira, V Pinho

**Affiliations:** 1 Laboratório de Resolução da Resposta Inflamatória, Departamento de Morfologia, Instituto de Ciências Biológicas, Universidade Federal de Minas Gerais, Belo Horizonte, Brazil; 2 Laboratório de Imunofarmacologia, Departamento de Bioquímica e Imunologia, Instituto de Ciências Biológicas, Universidade Federal de Minas Gerais, Belo Horizonte, Brazil; 3 Medical Research Council Centre for Inflammation Research, The Queen's Medical Research Institute, University of Edinburgh, Edinburgh, Scotland, UK; 4 Laboratório de Sinalização na Inflamação, Departamento de Análises Clínicas e Toxicológicas, Faculdade de Farmácia, Universidade Federal de Minas Gerais, Belo Horizonte, Brazil; 5 Departamento de Microbiologia, Instituto de Ciências Biológicas, Universidade Federal de Minas Gerais, Belo Horizonte, Brazil; 6 Laboratório de Inflamação, Instituto Oswaldo Cruz, FIOCRUZ, Rio de Janeiro, Brazil; 7 Laboratório de Patologia Geral, Instituto de Ciências Biológicas, Universidade Federal de Minas Gerais, Belo Horizonte, Brazil

## Abstract

Eosinophils are effector cells that have an important role in the pathogenesis of allergic disease. Defective removal of these cells likely leads to chronic inflammatory diseases such as asthma. Thus, there is great interest in understanding the mechanisms responsible for the elimination of eosinophils from inflammatory sites. Previous studies have demonstrated a role for certain mediators and molecular pathways responsible for the survival and death of leukocytes at sites of inflammation. Reactive oxygen species have been described as proinflammatory mediators but their role in the resolution phase of inflammation is poorly understood. The aim of this study was to investigate the effect of reactive oxygen species in the resolution of allergic inflammatory responses. An eosinophilic cell line (Eol-1) was treated with hydrogen peroxide and apoptosis was measured. Allergic inflammation was induced in ovalbumin sensitized and challenged mouse models and reactive oxygen species were administered at the peak of inflammatory cell infiltrate. Inflammatory cell numbers, cytokine and chemokine levels, mucus production, inflammatory cell apoptosis and peribronchiolar matrix deposition was quantified in the lungs. Resistance and elastance were measured at baseline and after aerosolized methacholine. Hydrogen peroxide accelerates resolution of airway inflammation by induction of caspase-dependent apoptosis of eosinophils and decrease remodeling, mucus deposition, inflammatory cytokine production and airway hyperreactivity. Moreover, the inhibition of reactive oxygen species production by apocynin or in gp91^phox−/−^ mice prolonged the inflammatory response. Hydrogen peroxide induces Eol-1 apoptosis *in vitro* and enhances the resolution of inflammation and improves lung function *in vivo* by inducing caspase-dependent apoptosis of eosinophils.

Eosinophils express numerous receptors and secrete a wide variety of inflammatory mediators that influence many innate and adaptive immune responses. These multifunctional cells are important in the defense against helminth infection and are involved in the pathogenesis of many eosinophilic dominant allergic diseases.^1^ High levels of eosinophil granule proteins (such as major basic protein (MBP)) have been found in bronchoalveolar lavage fluid from patients with asthma and evidence indicates that high-concentration granule products have contributed to the development of airway hyperreactivity (AHR), a cardinal feature of asthma.^2^ Asthma is an inflammatory disease of the airways with participation of many cell types including leukocytes especially eosinophils and lymphocytes.^3, 4^ Activation of these cells (mainly lymphocytes) leads to the release of proinflammatory mediators and cytokines such as leukotriene B_4_, interleukin-4 (IL-4), interleukin-5 (IL-5), interleukin-9 (IL-9), interleukin-13 (IL-13) and colony-stimulating factor granulocyte-macrophage (GM-CSF).^3, 5, 6, 7^ Investigations using preclinical animal models of asthma and clinical studies in patients with asthma have demonstrated that the presence of eosinophils in the lungs are associated with epithelial damage, goblet cell hyperplasia, smooth muscle hypertrophy and airway hyperresponsiveness resulting in airflow limitation which can be fatal.^3, 8, 9, 10^ Recently, anti-IL-5 treatment has been shown to ameliorate lung function in patients with eosinophilic asthma.^11^

Apoptosis of leukocytes is regarded as an important process for the successful resolution of inflammatory responses. Reduced eosinophil apoptosis in bronchoalveolar lavage (BAL) fluid has been shown to correlate positively with severity of asthma.^3, 12, 13, 14^ Indeed, defective leukocyte apoptosis and subsequent removal of apoptotic cells by phagocytes is thought to be important for the initiation and propagation of chronic inflammatory diseases such as asthma.^15^ Therefore, a balance in the tissue microenvironment between pro- and antiapoptotic signals is likely to greatly influence the load of eosinophils in the asthmatic lung.^16^ Thus, there is a great interest in understanding the mechanisms responsible for the elimination of eosinophils and other leukocytes and inactivation of proinflammatory mediators in inflammatory sites.^17^

Several molecular pathways have been shown to modulate the survival and death of leukocytes at sites of inflammation, including reactive oxygen species (ROS).^18^ ROS are a family of molecules containing oxygen and includes hydrogen peroxide (H_2_O_2_), superoxide O_2_^−^, hydroxyl radical (OH) and nitric oxide (NO).^19^ In inflammatory conditions, ROS are increased as they help in neutralizing invading organisms during infection either directly or indirectly by formation of extracellular traps (ETs).^20^ ROS have traditionally been regarded as quintessentially proinflammatory. However, evidence for ROS-mediated anti-inflammatory actions has been described.^21^ The importance for ROS production in the context of infection can be exemplified in patients with chronic granulomatous disease (CGD) where defective production in ROS results in multiple infections and often early death.^22, 23^ Furthermore, studies in mouse models have shown that NADPH oxidase is key for regulating lung inflammation and injury as well as NF-*κ*B activation and downstream cytokine production in response to LPS.^24^ More recently, our group has demonstrated that NADPH oxidase-derived H_2_O_2_ is directly linked to induction of apoptosis of neutrophils and resolution of inflammation in a model of antigen-induced arthritis.^18^ However, the role of ROS in the context of the resolution of allergic inflammation is still unknown.

Here, we evaluated whether H_2_O_2_ drives apoptosis of eosinophils and thereby influences the resolution of established eosinophilic inflammation and reduction of airflow obstruction. Our study provides evidence that H_2_O_2_ is released during allergic inflammation in a gp91^phox−/−^-dependent manner and induces a caspase-dependent proapoptotic effect in eosinophils, thus having a crucial role in the resolution of allergic inflammation.

## Results

### Kinetics of inflammation response after asthma induction in mice

We used a well-established murine model of asthma previously described by Stock *et al.*^25^ In this model, the inflammatory response was characterized by accumulation of leukocytes detectable at 12 h and that reached maximal at 24–48 h after challenge with OVA (Supplementary Figure 1A). There was predominant accumulation of neutrophils at 12 h after antigenic challenge (Supplementary Figure 1B). Twenty-four hours after challenge, the number of neutrophils dropped and there was increased accumulation of eosinophils. The natural resolution of eosinophilic inflammation was initiated at 48 h and virtually completed at 72 h after antigen challenge (Supplementary Figure 1C). Likewise, similar results were obtained in an allergic pleurisy model in which there is also acute self-resolving eosinophilic inflammation (Supplementary Figure 2A). Importantly, the increased number of mononuclear cells at 12 h and 48 h after antigen challenge coincided with the decrease of neutrophils and eosinophils, respectively (Supplementary Figure 1D).

### Treatment with H_2_O_2_ or SOD decreases eosinophil accumulation in asthma models

**Figure 1:**
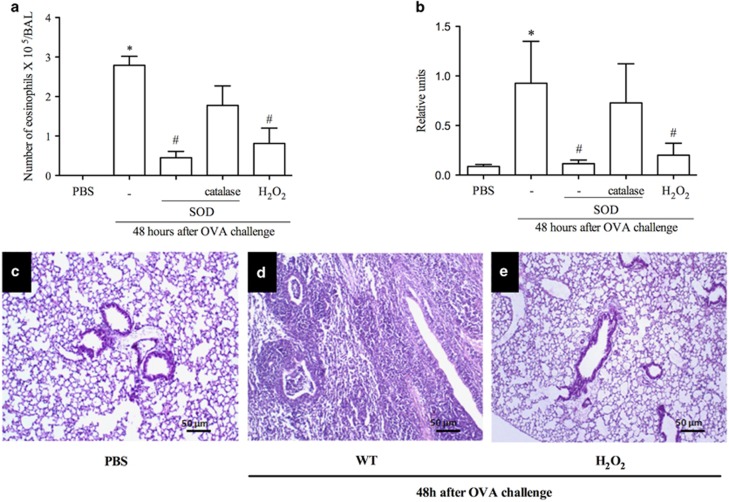
H_2_O_2_ promotes resolution of inflammation in a mouse model of asthma. Number of eosinophils after administration of SOD (0.3 mg/kg), SOD+catalase (1.2 mg/kg), H_2_O_2_ (0.5 M) or vehicle (30 *μ*l PBS) (**a**). Eosinophil peroxidase (**b**). Photomicrographs of lung sections - PBS (**c**), asthma (**d**) H_2_O_2_ (**e**) H&E. 100 × . Scale 50 *μ*m. Data represent mean±S.E.M. (*n*=5). *P<0.05 *versus* control mice. ^#^*P*<0.05 versus OVA mice

Next experiments were designed to investigate the ability of H_2_O_2_ to resolve eosinophilic inflammation *in vivo*. We treated mice with SOD or H_2_O_2_ 24 h after OVA challenge, a time point at which the number of neutrophils is close to basal levels and eosinophil numbers are maximal. Treatment with SOD, which increases production of endogenous H_2_O_2_, or exogenous H_2_O_2_ treatment decreased the number of eosinophils in the BAL (Figure 1a) and reduced the levels of eosinophilic peroxidase (EPO) in the lung (Figure 1b). Treatment with catalase, which degrades H_2_O_2_, prevented the proresolving effects of SOD (Figures 1a and b). The lung parenchyma of challenged mice exhibited intense infiltration of inflammatory cells and loss of pulmonary architecture 48 h after antigen challenge when compared with the control group (PBS) (Figures 1c and d). Mice that received H_2_O_2_ at 24 h showed reduced parenchymal inflammation (Figure 1e). Likewise, treatment with SOD was associated with less parenchymal inflammation and administration of catalase prevented the anti-inflammatory actions of SOD (data not shown). Likewise, similar results were obtained in an allergic pleurisy model (Supplementary Figures 2B and C).

### gp91^phox^ deficiency extends the duration of allergic inflammation

**Figure 2:**
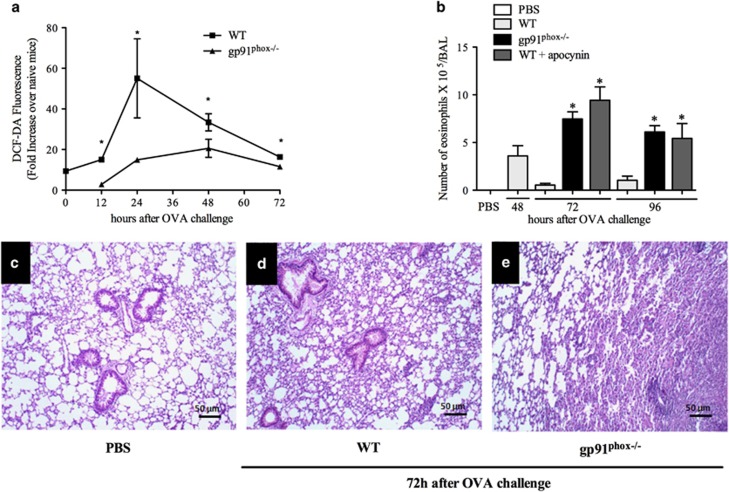
ROS derived from NADPH oxidase is necessary for inflammation resolution. ROS production in WT or gp91^phox−/−^ mice (**a**). Eosinophils 72 and 96 h after OVA in gp91^phox−/−^mice and after apocynin treatment (**b**). Eosinophil peroxidase (**c**). Lung sections of PBS (**d**), WT (**e**) gp91^phox−/−^(**f**). H&E. 100 × . Scale 50 *μ*m. Data represent mean±S.E.M. (*n*=5). *P<0.05 versus control group

Because treatment with H_2_O_2_ resolved eosinophilic inflammation, a series of experiments were then performed in gp91^phox−/−^ mice, which lack the ability to assemble the NADPH oxidase and have decreased capacity to generate ROS.^18^ ROS production in gp91^phox−/−^ mice was significantly lower when compared to wild-type mice (Figure 2a). In these mice, natural resolution of eosinophilic inflammation, which usually is complete within 72 h in WT mice, was not observed even at 96 h after antigen challenge (Figure 2b). Likewise, intranasal treatment with apocynin (an inhibitor of NADPH oxidase) at a dose of 10 mg/kg prevented natural resolution of eosinophilic inflammation at 72 and 96 h after challenge (Figure 2b).

The histopathological analysis of lung sections showed that within 72 h the inflammation was present only in focal segments in wild-type mice (WT) and lung tissue was mostly preserved (Figure 2d). In contrast, there was much infiltration of inflammatory cells and derangement of tissue architecture at 72 h in gp91^phox−/−^ mice exposed to antigen when compared with the control group (PBS, Figures 2d and e).

### H_2_O_2_ treatment induced caspase-dependent apoptosis of eosinophils

**Figure 3:**
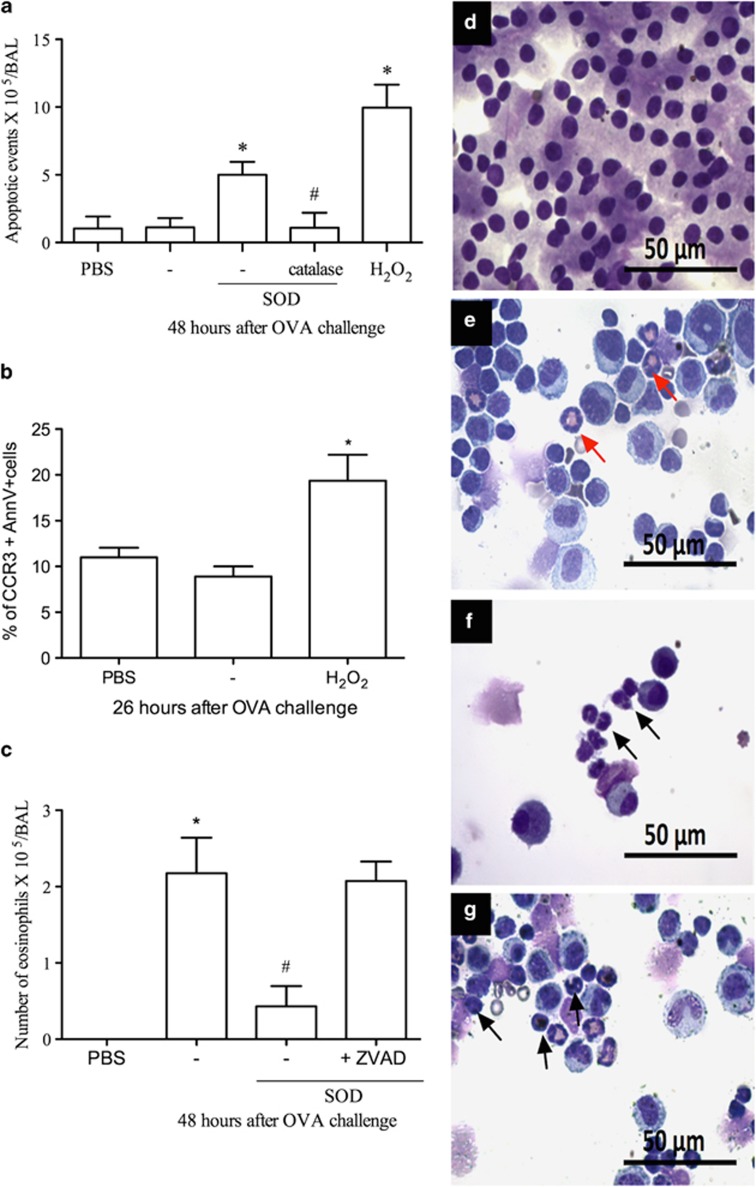
H_2_O_2_ induces apoptosis of eosinophils. SOD (0.3 mg/kg), SOD+catalase (1.2 mg/kg), H_2_O_2_ (0.5 M) or vehicle (30 *μ*l PBS) i.n. Apoptotic morphology (**a**) Cells expressing Annexin V-FITC+CCR3+ (**b**). Eosinophils after zVAD-fmk administration (1 mg/kg, i.p) (**c**). Cell types. Red arrow normal eosinophil. Black arrow: eosinophil with apoptotic morphology. PBS (**d**) asthma (**e**) SOD (**f**) H_2_O_2_ (**g**). 100 × Scale: 50μm. Data represent mean±S.E.M. (*n*=5). *P<0.05 versus control mice. ^#^*P*<0.05 *versus* OVA mice

Considering that the generation of endogenous H_2_O_2_ (via treatment with SOD) and administration of H_2_O_2_ reduced the number of eosinophils, we investigated whether death of eosinophils via apoptosis could underlie the resolution of eosinophilic inflammation. Indeed, there was significant increase in the number of apoptotic eosinophils as observed morphologically in mice treated with either SOD or H_2_O_2_ (Figures 3a and d–g and Supplementary Figure 2D) and demonstrated by flow cytometric analysis (Figure 3b). In mice treated with H_2_O_2_, there was an increase in expression of annexin V-FITC+ in CCR3+ cells at 2 h after treatment (Figure 3b). These data were confirmed by increased staining for cleaved caspase-3 in eosinophils, but not in mononuclear cells, in BAL from ova-challenged mice treated with H_2_O_2_ (Supplementary Figure 3). In contrast, T cells were not affected by H_2_O_2_ (% of cells CD3+/AnnX-V+: PBS: 0.69±0.24; OVA: 0.74±0.18, OVA+H_2_O_2_: 1.04±0.22 *P*>0.05, *n*=6), suggesting that H_2_O_2_ treatment affected eosinophils more specifically. Treatment of animals with zVAD-fmk, a pan-caspase inhibitor, reversed the proresolution actions of H_2_O_2_, demonstrating the caspase dependency of the resolution of eosinophilic inflammation (Figure 3c).

### H_2_O_2_ induces concentration-dependent apoptosis of an eosinophilic cell line *in vitro*

**Figure 4:**
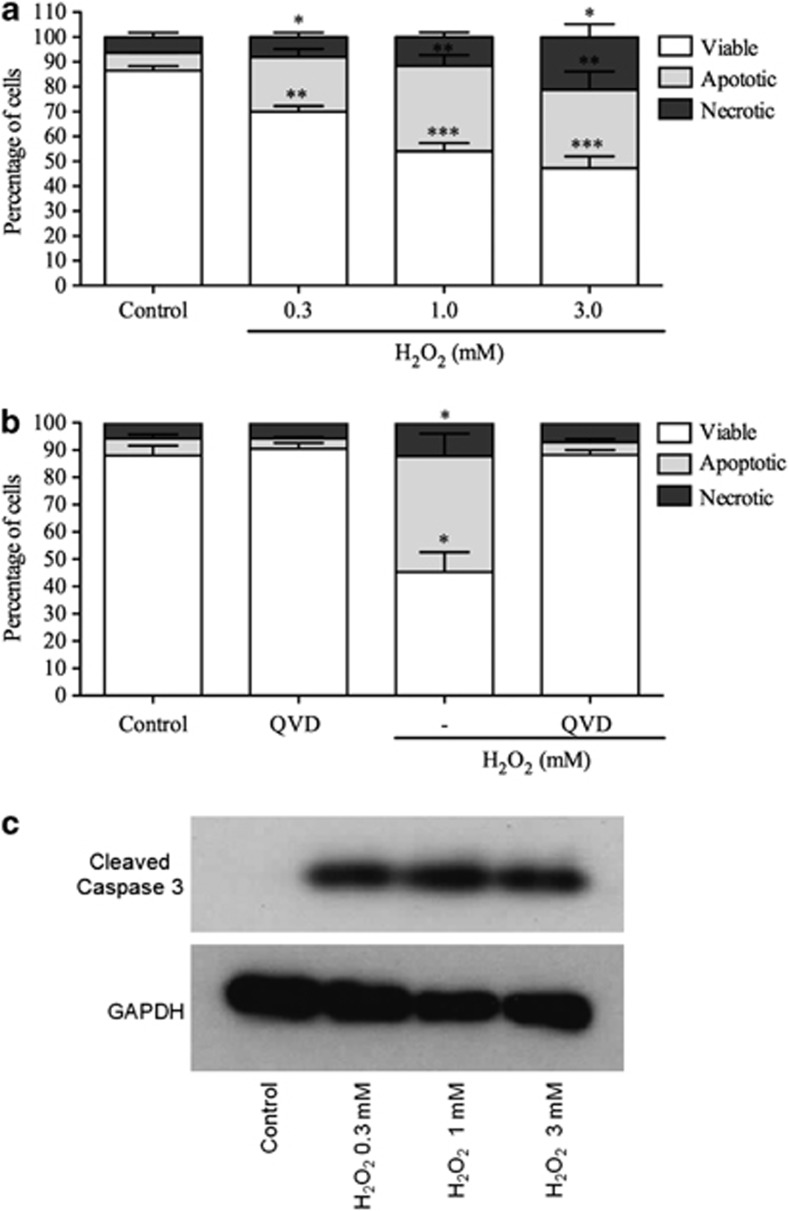
H_2_O_2_ induces concentration and caspase-dependent apoptosis in Eol-1 cells. Eol-1 cells with H_2_O_2_ (0.3 mM–3.0 mM) (**a**) or H_2_O_2_ (1 mM) and Q-VD-OPh (10 *μ*M) (**b**). Western blotting for cleaved caspase-3 (17/19 kDa) and GAPDH (37 kDa) (**c**). Data represent mean±S.E.M. *n*=3. *P<0.05, **P<0.01, ***P<0.001. Western blots representative on three experiments

Next, we evaluated whether H_2_O_2_ could induce directly the apoptosis of a human eosinophil cell line, Eol-1. This cell line has been extensively used as a model of eosinophil function, including chemotaxis, mediator release and apoptosis induction.^26, 27^ Eol-1 cells were incubated for a 4-h period with increasing concentrations of 0.3, 1.0 and 3.0 mM H_2_O_2_. Annexin-V-positive cells were considered apoptotic cells and annexin-V/PI dual-positive cells were considered late apoptotic/secondarily necrotic cells. H_2_O_2_ markedly increased apoptosis of Eol-1 cells in a concentration-dependent manner (Figure 4a). In order to confirm that Eol-1 cells undergo apoptosis following incubation with H_2_O_2_, Eol-1 was coincubated with H_2_O_2_ and Q-VD-OPh, a highly effective pan-caspase inhibitor. Coincubation of Eol-1 with Q-VD-OPh abrogated the ability of H_2_O_2_ to induce apoptosis (Figure 4b). To demonstrate further the role of caspase-3 in Eol-1 apoptosis induced by H_2_O_2_, we performed western blot analysis to investigate expression of cleaved caspase-3 on lysates from Eol-1 cells 4 h after stimulation with different concentrations of H_2_O_2_. H_2_O_2_ caused caspase-3 cleavage when compared with cells treated with media alone (Figure 4c). Apoptosis was also assessed morphologically using light microscopy after cytocentrifugation and staining with Diff-Quick. We observed that apoptotic Eol-1 cells exhibited nuclear condensation and cellular shrinkage, confirming flow cytometric data (data not shown).

### H_2_O_2_ reduces airway hyperreactivity to methacholine

**Figure 5:**
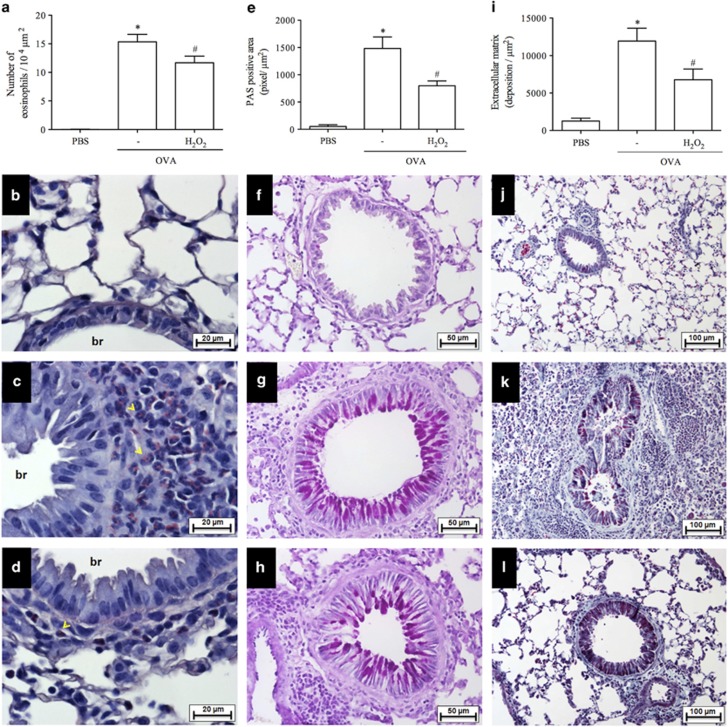
Effect of H_2_O_2_ on lung remodeling and mucus deposition. Leukocyte infiltrate around the bronchial region of the lung sections stained with hematoxylin and eosin (H&E) or Sirius Red (pH 10.2) (**a**–**d**). Mucus production from airway stained with hematoxylin and Periodic acid-Schiff (PAS) (**e**–**h**). 400 × . Peribronchial fibrosis stained with hematoxylin and eosin and a combination of Gomori trichrome (**i**–**l**). 200 × . *P<00.5 *versus* control mice. ^#^*P*<0.05 *versus* OVA mice

**Figure 6:**
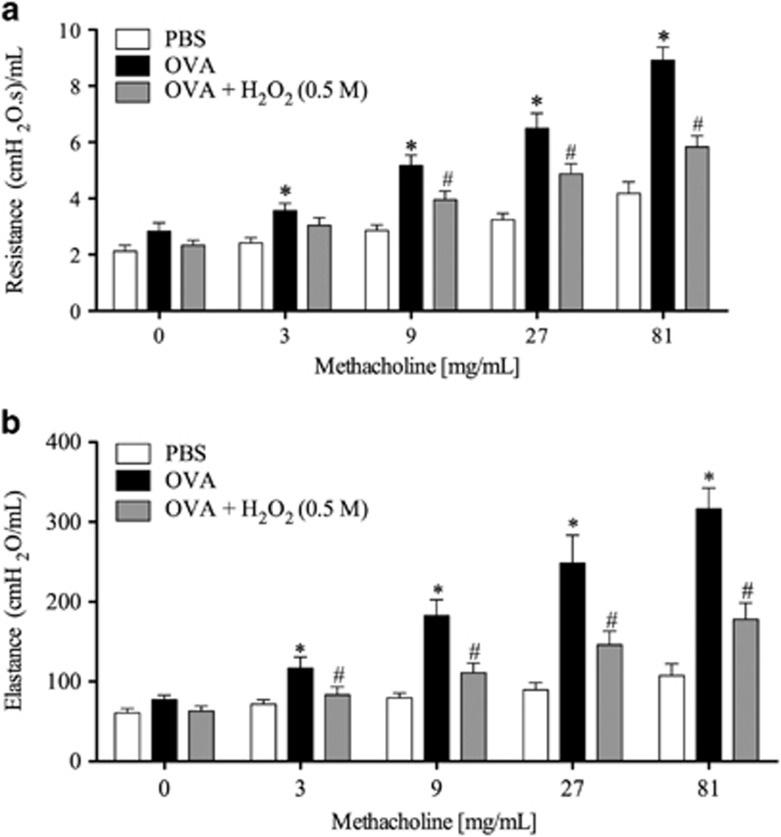
Airway resistance and dynamic elastance after increasing methacholine doses. H_2_O_2_ (0.5 M) or vehicle (30 *μ*l PBS) was administered i.n. instillation 24 h after asthma induction. Airway resistance (**a**) and elastance (**b**) was measured in response to increasing doses of methacholine (3, 9, 27, 81). Data represent mean ±S.E.M. (*n*=10). *P<0.05 *versus* vehicle group. ^#^*P*<0.05 *versus* OVA mice

Airway hyperreactivity (AHR) has been studied in a well-established murine model of asthma.^28^ In this model, the time course of the eosinophilic infiltrate was similar to that observed in our previous experiments (see Supplementary Figure 4 and compare with Supplementary Figure 1). Akin to experiments described previously, delayed treatment with H_2_O_2_ resolved the eosinophilic infiltrate in the BAL at 48 h after antigen challenge (Figures 5a and d) when compared with the control group (PBS, Figures 5a and b). In these mice, AHR was assessed by measuring airway resistance (RI) and lung elastance parameters in mechanically ventilated animals. As shown in Figure 6, antigen challenge with OVA exacerbated airway resistance and elastance of the lungs in response to inhaled methacholine (3–81 mg/ml), as compared with the control group challenged with PBS. Treatment with H_2_O_2_ reduced AHR, as seen by decreased airway resistance and elastance (Figure 6).

### Effect of H_2_O_2_ administration on mucus deposition and lung remodeling

To evaluate mucus production, sections of lung tissue were stained with periodic acid-Schiff. The analysis of airway mucins demonstrated significant metaplasia of goblet cells and mucus accumulation at 48 h after the last OVA challenge in actively sensitized mice (Figures 5e and g) in comparison with the control group (PBS) (Figure 5f). Treatment with H_2_O_2_ reduced OVA-induced mucus accumulation and the percentage of mucus producing cells to values similar to those observed in nonallergic controls (Figures 5e, f and h). The lung sections stained with Gomori trichrome demonstrated that OVA mice had increased peribronchiolar matrix deposition as compared with the PBS mice (Figures 5i–l). Quantitative analyses demonstrated that delayed treatment with H_2_O_2_ prevented extracellular matrix deposition in challenged mice (Figures 5i and l). Furthermore, H_2_O_2_ treatment decreased concentrations of CCL11, CCL24, IL-4, IL-5 and TNF-*α* that were measured at 48 h after antigen challenge (Table 1).

## Discussion

There is strong evidence indicating an important role for eosinophils in the pathogenesis of allergic diseases through the release of a variety of inflammatory mediators, including MBP, EPO and cytokines.^1^ Here, we evaluated the role of endogenous and effects of exogenous administration of H_2_O_2_ in the context of resolution of allergic inflammation. The results presented here can be summarized as follows: (i) treatment with H_2_O_2_ or strategies that enhanced H_2_O_2_ reduced eosinophil accumulation in the BAL and lung tissue; (ii) H_2_O_2_ promoted resolution of inflammation by inducing caspase-dependent apoptosis of eosinophils *in vivo*; (iii) H_2_O_2_ also induced caspase-dependent apoptosis of a human eosinophilic cell line *in vitro*. (iv) H_2_O_2_ derived from NADPH oxidase was necessary for natural resolution of allergic inflammation; (v) finally, treatment with H_2_O_2_ decreased secretion of mucus, extracellular matrix deposition, inflammatory cytokine production and decreased AHR induced by antigen. Altogether, these results demonstrate a clear proresolving effect of H_2_O_2_ in allergic inflammation *in vivo*.

In our experiments, we have demonstrated that endogenous or exogenous H_2_O_2_ resolved eosinophilic inflammation in two different models of the allergic response. In the asthma model, this event correlated with increased number of apoptotic eosinophils. This is similar to the role of H_2_O_2_ in a model of arthritis, in which H_2_O_2_ limits inflammation associated with induction of caspase-dependent apoptosis of neutrophils.^18^ In fact, blockade of caspases with a pan-caspase inhibitor, zVAD-fmk, prevented apoptosis and the resolution of eosinophilic inflammation induced by H_2_O_2_ consistent with our previous studies on neutrophils. H_2_O_2_ has been shown to induce apoptosis in culture of different cell types including human hepatocyte cells,^29^ epithelial cells,^30^ endothelial cells^31^ and myocytes.^32^ Here, we demonstrate that H_2_O_2_ also induced apoptosis of a human eosinophilic cell line, as by assessed by flow cytometry, light microscopy and cleavage of caspase-3 assessed by western blotting. The experiments using Eol-1 cells suggest that this pathway may be relevant in humans and clearly deserve further investigation in human disease. Indeed, ROS have been reported to increase apoptosis of human eosinophils.^33, 34^ Therefore, our evidence suggests that resolution of eosinophilic inflammation by endogenous or exogenous H_2_O_2_ is due to the capacity of this molecule to induce caspase-dependent apoptosis of eosinophils.

Results in gp91phox-deficient mice showed that influx was not altered but persistency of eosinophils in tissues was greatly prolonged. Moreover, the intranasal treatment with an inhibitor of NADPH oxidase delayed the resolution of inflammatory response. It has been reported that in conditions of oxidative stress, the NADPH oxidase complex catalyzes electron transfer from NADPH to molecular oxygen and generates superoxide anions (O_2_^−^). The superoxide dismutase (SOD) degrades O_2_^−^ in H_2_O and produces H_2_O_2_.^23^ Our work is the first to describe the importance of the oxidative pathway involving NADPH oxidase to resolve eosinophilic response. This is consistent with other studies in mice, which showed that gp91^phox−/−^ mice had delayed resolution of neutrophilic inflammation and this process was reversed by administration of exogenous H_2_O_2_ in a model of antigen-induced arthritis.^18^ These data support the concept that ROS, which are generated by the phagocytic NADPH oxidase and commonly considered harmful mediators of acute inflammation, have a role in limiting inflammation and may be pivotal in resolving acute inflammation.^35^ Indeed, our results show that H_2_O_2_ not only induces resolution of inflammation, but it is also relevant in the context of the natural resolution of eosinophilic inflammation. Recent data show that administration of SOD accelerates resolution of inflammation associated to antigen-induced arthritis resulting in increased number of apoptotic neutrophils. This coincided with activation of caspase-3 and increased Bax expression in neutrophils recovered from the articular cavity.^18^ We also investigated whether reactive nitrogen species (RNS) participate in the resolution of the inflammatory response in the model of allergic asthma. It was observed that in the absence of NO production by iNOS there was no change in the resolution of allergic inflammation (Supplementary Figure 5). RNS such as NO are involved in the inflammatory process in the airways of asthmatic patients. NO has also been shown to be involved in the regulation of apoptosis: it can prevent or induce apoptosis depending upon the cell type and the concentration in which it is produced.^36, 37, 38, 39, 40^ The data suggest that RNS are not essential for resolution of allergic inflammation.

Our results showed that in addition to resolving eosinophilic inflammation, treatment with H_2_O_2_ had major physiological consequences in a model of allergic asthma. Indeed, treatment with H_2_O_2_ greatly reduced eosinophil accumulation, changes in airway reactivity, remodeling and mucus deposition induced by antigen challenge of immunized mice. Cytokines, including IL-4, IL-5 and TNF-*α*, and chemokines (CCL11 and CCL24) are thought to be involved in eosinophil accumulation and linked to pathophysiology of allergic disease.^41, 42, 43, 44^ There is also evidence that IL-5 and TNF-α promote eosinophil survival *in vitro.*^45, 46, 47^ In addition, CCL11 and CCL24 are important for eosinophil recruitment from the blood to the parenchyma and then into the airway.^48^ However, the evidence that antibody or drugs targeting these cytokines or chemokine induce resolution of eosinophilic inflammation *in vivo* is scanty. In addition, IL-4, IL-5 and TNF-α are essential for the development of AHR, mucus production and fibrosis in asthma.^49, 50, 51^ Similarly, chemokines, such as CCL11 and CCL24, may also contribute to AHR and fibrogenesis in animal models of asthma.^42, 48^ In our studies, there was a marked decrease in levels of these cytokines, which accompanied the resolution of the eosinophil numbers. Resolution of inflammation is known to exert potent anti-inflammatory effects and decrease of production of proinflammatory cytokines,^52, 53^ suggesting that decreased levels of TNF, IL-5, IL-4 and chemokines are likely a consequence of the anti-inflammatory action of the resolution process. Alternatively, it is also possible that ROS induced a primary decrease of cytokines that could have then accounted for eosinophil apoptosis and in the resolution of eosinophilic inflammation. The latter possibility is more difficult to ascertain in the *in vivo* situation and clearly more studies are needed to convincingly show that blockade of cytokines alter survival of eosinophils *in vivo*, as demonstrated *in vitro*. Whatever the mechanism, direct effect on eosinophils (shown here) or an indirect action via decrease on survival factors, our data clearly demonstrate the proresolving effects of H_2_O_2_ in the context of eosinophilic inflammation.Taken together our findings show that exogenous or endogenous generation of H_2_O_2_ resolve allergic inflammation by inducing eosinophil apoptosis in a caspase-dependent manner. Induction of eosinophil apoptosis by H_2_O_2_ decreases airway remodeling and dysfunction. These results have fundamental implications to the basic concept of inflammation resolution and may have therapeutic implications. Indeed, our results demonstrate that resolution of eosinophilic inflammation by H_2_O_2_ maintains tissue integrity and function. Restoration of tissue inflammation to homeostasis may reverse airway function and remodeling in patients, a tenet that may be exploited for the development of novel therapies for the treatment of asthma.

## Materials and Methods

### Ethics statement

Male C57/BL6 and gp91^phox−/−^ mice (Gene *Cybb;* ES Cell Line name CCE/EK.CE)^54^ (8–10 weeks) were bred and housed in a temperature-controlled room with free access to water and food. Animal Care and Use Committee and the study received prior approval from the local animal ethics committee (Animal Ethics Review Board – Comitê de Ética em Experimentação Animal-CETEA/Universidade Federal de Minas Gerais-UFMG (protocol number: 218/11).

### Reagents and drugs

Superoxide dismutase (SOD) from bovine erythrocytes, catalase, H_2_O_2_, ovalbumin, Z-VAD-fmk, propidium iodide (PI) and 4,5-diaminofluoresceína-diacetato (DAF-2DA) were purchase from Sigma (St. Louis, MO, USA). Apo DETECT ANNEXIN-V-FITC KIT 2,7-diclorodihidrofluoresceína-diacetato (DCF-DA) and dihidrorodamine-123 (DHR-123) were purchase from Invitrogen (Life Technologies, São Paulo, Brazil). Antibodies were purchased from Santa Cruz Biotechnology (Santa Cruz, CA, USA). Q-VD-OPh (R&D Systems, Minneapolis, MN, USA), annexin-V-FLUOS (Roche, Mannheim, Germany).

### Eol-1 cell culture and apoptosis induction

Human eosinophilic cell line (Eol-1 cells) was maintained in RPMI-1640 medium (PAA) with 2% FBS (Biosera), penicillin (100 U/ml) and streptomycin (100 U/ml) (PAA). Cells were aliquoted (2 × 10^6^ cells/ml) and incubated with H_2_O_2_ (Thermo Fisher Scientific, Waltham, MA, USA), Q-VD-OPh (R&D Systems) or combinations of these either in 96-well-flat bottomed-plates (final volume of 150 *μ*l) or in 2 ml Eppendorf tubes (final volume of 500 *μ*l) in a humidified, 37 °C incubator at 5% CO_2_ atmosphere or on a shaking, temperature-controlled heat block. Q-VD-OPh stock was initially dissolved in dimethyl sulphoxide (Sigma) then diluted in buffer yielding a final concentration of 0.2% a corresponding DMSO control of 0.2% was assessed as an appropriate vehicle control. Apoptosis was assessed by flow cytometry with a BD-LSR Fortessa (Becton Dickinson Biosciences, San Jose, CA, USA) using annexin-V-FLUOS (Roche) in combination with propidium iodide (PI) (Sigma) as described.^55^ Data were analyzed using Flowjo software (TreeStar, Ashland, OR, USA). Morphological apoptotic changes were assessed by light microscopy of DiffQuick stained cytocentrifuged cells.^55^

### Western blotting

Cells at a concentration of 2 × 10^6^ cells/ml per condition were incubated with H_2_O_2_ (Thermo Fisher Scientific), Q-VD-OPh (R&D Systems) or combinations of these at 37 °C on a shaking heat block for 4 h. Eol-1 cells were pelleted by centrifugation at 3000 × *g* for 60 s and resuspended with whole-cell lysis buffer. Sample was incubated on ice for 10 min then NP-40 was added, briefly vortexed and centrifuged for 20 min at 3000 × *g*. Supernatant was removed and the remaining cell pellet was resuspended in sample buffer before boiling at 95 °C for 5 min. Lysate were run on 12% precast gels (Thermo Fisher Scientific, Rockford, IL, USA) and transferred onto PVDF (Immobilon-P, Millipore, Herts, UK). Membranes were blocked for 1 h in 5% (wt/vol) dried milk/TBS/0.1% Tween-20 before probing with antibodies to cleaved caspase-3 diluted 1 : 500 (Cell Signaling Technologies) at 4^0^C overnight or GAPDH diluted 1 : 20 000 (Sigma-Aldrich, St. Louis, MO, USA) 1 h at room temperature. Following 3 × 5 min washes in TBS/0.1%Tween-20, the blots were incubated with HRP-conjugated secondary antibody (Dako, Glostrup, Denmark) diluted 1 : 2500 for 1 h at room temperature before incubation with ECL (GE Healthcare, Bucks, UK) exposure to BioMax MS-1 X-ray-sensitive film, and processing (X-Ograph Imaging Systems, Wilts, UK).

### Induction of asthma

All mice were sensitized intraperitoneally (i.p.) with 100 *μ*g of OVA (albumin from chicken egg white - A5503, Sigma-Aldrich) in 2% alum (aluminum hydroxide gel adjuvant; Brenntag) on day 0, then challenged intranasally (i.n.) on days 8–10 with 10 *μ*g of OVA or PBS.^56^ The treatments with apocynin, SOD, H_2_O_2_ and catalase were performed i.n. and the zVAD-fmk (Tocris Bioscience) was administered i.p.

### Induction of pleurisy

Mice were immunized with OVA adsorbed to aluminum hydroxide gel as described.^52^ Briefly, mice were injected subcutaneously (s.c.) on days 1 and 7 with 0.2 ml of a solution containing 100 mg of OVA and 70 mg of aluminum hydroxide. Sensitized mice were then challenged with OVA (1 mg/cavity, in a total volume of 100 ml intrapleurally, i.pl) or PBS. Cells present in the pleural cavity were collected at different times by washing the cavity with 2 ml PBS and total cell counts performed in a modified Neubauer chamber using Turk's stain. Differential cell counts were performed on cytocentrifuge preparations (Shandon Cytospin III), stained with May–Grünwald–Giemsa using standard morphological criteria to identify cell types. The results are presented as the number of cells/cavity.

### BAL analysis

BAL was performed to obtain leukocytes present in the alveolar space. Mice were killed by anesthetic overdose and the trachea of each animal was exposed and cannulated with a polypropylene catheter of 1.7 mm. Airways were washed with 2 ml of ice-cold PBS. Total cell counts were performed in a modified Neubauer chamber using Turk's stain. Differential cell counts were performed on cytocentrifuge preparations (Shandon Cytospin III), stained with May–Grünwald–Giemsa using standard morphological criteria to identify cell types. The results are presented as the number of cells/BAL. In a separated set of experiments, apoptotic cells were morphologically identified in cytocentrifuged slides, which were also positively stained for cleaved caspase-3 (Alexa Fluor 488 rabbit anti-mouse cleaved caspase-3; Cell Signaling; 1 : 50). Fluorescence intensity was measured offline using Volocity software 6.3 (Perkin–Elmer, Waltham, MA, USA) and fluoresce profile was assessed using Image J (NIH).^57^

### Histological analysis

The lung was prepared as described.^58^ Briefly, lungs were removed 24 h after the last challenge and fixed in Milloning buffer solution (pH 7.4) with 4% paraformaldehyde. For analysis of leukocyte infiltrate around the bronchial region, the lung sections were stained with hematoxylin and eosin (H&E) or were subjected to Sirius Red (pH 10.2) staining (Llewellyn's Sirus Red Direct Red 80, CI 35780; Aldrich, Milwaukee, WI, USA). Results were expressed as leukocytes/10^4^ *μ*m^2^. Mucus production was analyzed from tissue sections stained with Harris hematoxylin stain and a combination of Periodic acid-Schiff (PAS) stain (Schiff's reagent, Merck, Rio de Janeiro, Brazil). Photomicrographs of airways obtained at 400 × magnification were analyzed using the software Image-Pro Plus (Image-Pro Plus, 4.1; Media Cybernetics, Houston, TX, USA). Nine to twelve bronchial areas per lung were outlined and quantified. Results were expressed as PAS positive area (pixels/*μ*m^2^). Peribronchial fibrosis was analyzed from tissue sections stained with hematoxylin and eosin stain and a combination of Gömöritrichrome stain (Trichrome Stain LG Solution; Sigma-Aldrich). Photomicrographs of airways obtained at 200 × magnification were analyzed using the software Image-Pro Plus. Eight to twelve peribronchial area per lung were outlined and quantified.^59^ Results were expressed as extracellular matrix deposition area (*μ*m^2^).

### Assessment of leukocyte apoptosis

Apoptosis was assessed as described.^52, 60^ Briefly, cells (5 × 10^4^) collected 48 h after antigen challenge were cytocentrifuged, fixed and stained with May–Grunwald–Giemsa and counted using oil immersion microscopy ( × 100 objective) to determine the proportion of cells with distinctive apoptotic morphology (cell shrinkage, chromatin condensation, nuclear fragmentation and maintenance of membrane integrity). Twenty-five fields were counted per slide and the results expressed as the mean±S.E.M. of number of apoptotic cells in 25 fields. Assessment of apoptosis was also performed by flow cytometry using commercial kit annexin-V-FITC (Invitrogen) following the instructions of the manufacturer in cells previously stained with the following Abs to extracellular markers for 30 min on ice: anti-mouse CCR3 mAb (BD Biosciences, San Jose, CA, USA) or anti-mouse mAb CD3 (BD Biosciences). Flow cytometry was performed using a FACS Canto flow cytometer (BD). At least 10^4^ events were recorded and analyzed using FlowJo software (FlowJo, LLC, Ashland, OR, USA). Results are expressed as cells undergoing early stage apoptosis quantified by staining with annexin-V but not PI.

### ELISA analysis

Murine IL-4, IL-5, TNF-*α*, CCL11 and CCL24 levels were measured in right lung tissue samples by means of ELISA technique using commercial DuoSet kits R&D Systems following the instructions of the manufacturer. Results were expressed in levels of cytokines per lung (pg/ml).

### Quantification of eosinophil accumulation in lung

Pulmonary EPO activity was determined to estimate eosinophil recruitment into the lung parenchyma as described.^61^ Absorbance was read in an ELISA reader (Expert Plus ASYS Hitech GmbH, Eugenorf, Austria) at 492 nm. Values are expressed in O.D.

### Determination of the production of reactive oxygen species and nitrogen by fluorimetry

Leukocytes obtained from the BAL were incubated separately with the probes '-7' dicloro-dihidrofluoesceínadiacetate (DCF-DA 20 uM), dihidrorodaminadiacetate 123 (DHR-123 5 uM) of 4,5-diaminofluorescein (DAF-2DA 10 uM) for 30 min in an oven at 37 °C. This stage of the experiment was performed in the dark, because the markers are photosensitive. The reading of fluorescence was performed in a fluorescence spectrophotometer (Synergy 2, Biotek, Winooski, VT, USA) with wavelengths of excitation and emission of 488 and 515 nm, respectively.

### Invasive assessment of respiratory mechanics

Airway reactivity was assessed as a change in airway function after challenge with aerosolized methacholine in a FinePoint R/C Buxco Platform. The parameters were measured as previously described.^62^ The analyses were performed 48 h after the last OVA challenge.

### Statistical analysis

ANOVA followed by Student Newman–Keuls was applied to comparison of multiple groups. In order to test statistical significance between two groups we used the unpaired Student's *t*-test (GraphPad Software, San Diego, CA, USA). All *in vitro* experiments were performed at least three times with each experiment carried out in triplicate. All *in vivo* experiments included six mice per group. Data were expressed as the mean ±S.E.M. Differences were considered significant at *P*<0.05.

## Figures and Tables

**Table 1:** Levels of cytokines in lung (pg/ml)

**Cytokines**	**PBS**	**OVA**	**H_2_O_2_**
CCL11	743±152	9109±5063^a^	5845±2048^b^
CCL24	903±245	11956±6304^a^	6896±1143^b^
IL-4	110±32	238±149^a^	127±34^b^
IL-5	847±84	1226±445^a^	829±140^b^
TNF-*α*	182±41	310±135^a^	157±45^b^

Abbreviations: TNF-*α*, tumor necrosis factor alpha; IL-4, interleukin-4; IL-5, interleukin-5

a Significantly different from the control group (*P*<0.05)

b Significantly different from the vehicle group (*P*<0.05)

## Abbreviations

CAT: catalase
SOD: superoxide dismutase
MBP: eosinophil granule proteins
AHR: airway hyperreactivity
ROS: reactive oxygen species
RNS: Reactive nitrogen species
OVA: ovalbumin
EPO: eosinophil peroxidase

## References

[bib1] 1Luna-Gomes T, Bozza PT, Bandeira-Melo C. Eosinophil recruitment and activation: the role of lipid mediators. Fronti Pharmacol 2013; 4: 27. 10.3389/fphar.2013.00027 PMC3605515 23525348

[bib2] 2Rothenberg ME, Hogan SP. The eosinophil. Ann Rev Immunol 2006; 24: 147–174. 16551246 10.1146/annurev.immunol.24.021605.090720

[bib3] 3Holgate ST. The airway epithelium is central to the pathogenesis of asthma. Allergol Int 2008; 57: 1–10. 18209502 10.2332/allergolint.R-07-154

[bib4] 4Felton JM, Lucas CD, Rossi AG, Dransfield I. Eosinophils in the lung - modulating apoptosis and efferocytosis in airway inflammation. Front Immunol 2014; 5: 302. 25071763 10.3389/fimmu.2014.00302 PMC4076794

[bib5] 5Kay AB. The role of T lymphocytes in asthma. Chem Immunol Allergy 2006; 91: 59–75. 16354949 10.1159/000090230

[bib6] 6Afshar R, Medoff BD, Luster AD. Allergic asthma: a tale of many T cells. Clin Exp Allergy 2008; 38: 1847–1857. 19037961 10.1111/j.1365-2222.2008.03119.x

[bib7] 7Nouri-Aria KT, Durham SR. Regulatory T cells and allergic disease. Inflamm Allergy Drug Targets 2008; 7: 237–252. 19075789 10.2174/187152808786848405

[bib8] 8Jacobsen EA, Helmers RA, Lee JJ, Lee NA. The expanding role(s) of eosinophils in health and disease. Blood 2012; 120: 3882–3890. 22936660 10.1182/blood-2012-06-330845 PMC3496950

[bib9] 9Fulkerson PC, Rothenberg ME. Targeting eosinophils in allergy, inflammation and beyond. Nat Rev Drug Discov 2013; 12: 117–129. 23334207 10.1038/nrd3838 PMC3822762

[bib10] 10Wilson SJ, Rigden HM, Ward JA, Laviolette M, Jarjour NN, Djukanovic R. The relationship between eosinophilia and airway remodelling in mild asthma. Clin Exp Allergy 2013; 112: 1342–1350. 10.1111/cea.12156 PMC4040270 24261944

[bib11] 11Nair P. Anti-interleukin-5 monoclonal antibody to treat severe eosinophilic asthma. N Eng J Med 2014; 371: 1249–1251. 10.1056/NEJMe1408614 25197762

[bib12] 12Duncan CJA, Lawrie A, Blaylock MG, Douglas JG, Walsh GM. Reduced eosinophil apoptosis in induced sputum correlates with asthma severity. Eur Respir J 2003; 22: 484–490. 14516139 10.1183/09031936.03.00109803a

[bib13] 13Walsh GM. Eosinophil apoptosis: Mechanisms and clinical relevance in asthmatic and allergic inflammation. Brit J Haematol 2000; 111: 61–67. 11091183

[bib14] 14Vignola AM, Chanez P, Chiappara G, Siena L, Merendino A, Reina C et al. Evaluation of apoptosis of eosinophils, macrophages, and T lymphocytes in mucosal biopsy specimens of patients with asthma and chronic bronchitis. J Allergy Clin Immunol 1999; 103: 563–573. 10200002 10.1016/s0091-6749(99)70225-3

[bib15] 15Gautier EL, Ivanov S, Lesnik P, Randolph GJ. Local apoptosis mediates clearance of macrophages from resolving inflammation in mice. Blood 2013; 122: 2714–2722. 23974197 10.1182/blood-2013-01-478206 PMC3795463

[bib16] 16Walsh GM. Eosinophil apoptosis and clearance in asthma. J Cell Death 2013; 6: 17–25. 25278777 10.4137/JCD.S10818 PMC4147767

[bib17] 17Alessandri AL, Duffin R, Leitch AE, Lucas CD, Sheldrake TA, Dorward DA et al. Induction of eosinophil apoptosis by the cyclin-dependent kinase inhibitor AT7519 promotes the resolution of eosinophil-dominant allergic inflammation. PloS One 2011; 6: e25683. 21984938 10.1371/journal.pone.0025683 PMC3184151

[bib18] 18Lopes F, Coelho FM, Costa VV, Vieira EL, Sousa LP, Silva TA et al. Resolution of neutrophilic inflammation by H2O2 in antigen-induced arthritis. Arthritis Rheum 2011; 63: 2651–2660. 21567381 10.1002/art.30448

[bib19] 19Fialkow L, Wang Y, Downey GP. Reactive oxygen and nitrogen species as signaling molecules regulating neutrophil function. Free Radic Biol Med 2007; 42: 153–164. 17189821 10.1016/j.freeradbiomed.2006.09.030

[bib20] 20Wartha F, Henriques-Normark B. ETosis: a novel cell death pathway. Sci Signal 2008; 1: pe25. 18506034 10.1126/stke.121pe25

[bib21] 21Sareila O, Kelkka T, Pizzolla A, Hultqvist M, Holmdahl R. NOX2 complex-derived ROS as immune regulators. Antioxid Redox Signal 2011; 15: 2197–2208. 20919938 10.1089/ars.2010.3635

[bib22] 22Segal BH, Grimm MJ, Khan AN, Han W, Blackwell TS. Regulation of innate immunity by NADPH oxidase. Free Rad Biol Med 2012; 53: 72–80. 22583699 10.1016/j.freeradbiomed.2012.04.022 PMC3377837

[bib23] 23Gardiner GJ, Deffit SN, McLetchie S, Perez L, Walline CC, Blum JS. A Role for NADPH oxidase in antigen presentation. Front Immunol 2013; 4: 295. 24069023 10.3389/fimmu.2013.00295 PMC3779930

[bib24] 24Han W, Li H, Cai J, Gleaves LA, Polosukhin VV, Segal BH et al. NADPH oxidase limits lipopolysaccharide-induced lung inflammation and injury in mice through reduction-oxidation regulation of NF-kappaB activity. J Immunol 2013; 190: 4786–4794. 23530143 10.4049/jimmunol.1201809 PMC3633681

[bib25] 25Stock P, Akbari O, Berry G, Freeman GJ, Dekruyff RH, Umetsu DT. Induction of T helper type 1-like regulatory cells that express Foxp3 and protect against airway hyper-reactivity. Nat Immunol 2004; 5: 1149–1156. 15448689 10.1038/ni1122

[bib26] 26Noda H, Sakagami H, Kokubu F, Kurokawa M, Tokunaga H, Takeda M et al. Induction of apoptosis in human eosinophilic leukemic cell line (EOL-1). Int Arch Allergy Immunol 1997; 114(Suppl 1): 84–88. 9363935 10.1159/000237727

[bib27] 27Mayumi M. EoL-1, a human eosinophilic cell line. Leuk Lymphoma 1992; 7: 243–250. 1477652 10.3109/10428199209053629

[bib28] 28Bezerra-Santos CR, Vieira-de-Abreu A, Vieira GC, Filho JR, Barbosa-Filho JM, Pires AL et al. Effectiveness of Cissampelos sympodialis and its isolated alkaloid warifteine in airway hyperreactivity and lung remodeling in a mouse model of asthma. Int Immunopharmacol 2012; 13: 148–155. 22480776 10.1016/j.intimp.2012.03.014

[bib29] 29Kim SJ, Jung HJ, Hyun DH, Park EH, Kim YM, Lim CJ. Glutathione reductase plays an anti-apoptotic role against oxidative stress in human hepatoma cells. Biochimie 2010; 92: 927–932. 20302905 10.1016/j.biochi.2010.03.007

[bib30] 30Hussain S, Thomassen LC, Ferecatu I, Borot MC, Andreau K, Martens JA et al. Carbon black and titanium dioxide nanoparticles elicit distinct apoptotic pathways in bronchial epithelial cells. Part Fibre Toxicol 2010; 7: 10. 20398356 10.1186/1743-8977-7-10 PMC2873464

[bib31] 31Fang WT, Li HJ, Zhou LS. Protective effects of prostaglandin E1 on human umbilical vein endothelial cell injury induced by hydrogen peroxide. Acta Pharmacologica Sinica 2010; 31: 485–492. 20305680 10.1038/aps.2010.23 PMC4007667

[bib32] 32Zhang L, Jiang H, Gao X, Zou Y, Liu M, Liang Y et al. Heat shock transcription factor-1 inhibits H2O2-induced apoptosis via down-regulation of reactive oxygen species in cardiac myocytes. Mol Cell Biochem 2011; 347: 21–28. 20941531 10.1007/s11010-010-0608-1

[bib33] 33Kankaanranta H, Giembycz MA, Barnes PJ, Haddad el B, Saarelainen S, Zhang X et al. Hydrogen peroxide reverses IL-5 afforded eosinophil survival and promotes constitutive human eosinophil apoptosis. Int Arch Allergy Immunol 2002; 127: 73–78. 11893856 10.1159/000048171

[bib34] 34Wedi B, Straede J, Wieland B, Kapp A. Eosinophil apoptosis is mediated by stimulators of cellular oxidative metabolisms and inhibited by antioxidants: involvement of a thiol-sensitive redox regulation in eosinophil cell death. Blood 1999; 94: 2365–2373. 10498608

[bib35] 35Deng J, Wang X, Qian F, Vogel S, Xiao L, Ranjan R et al. Protective role of reactive oxygen species in endotoxin-induced lung inflammation through modulation of IL-10 expression. J Immunol 2012; 188: 5734–5740. 22547702 10.4049/jimmunol.1101323 PMC3358534

[bib36] 36Zhang X, Moilanen E, Lahti A, Hamalainen M, Giembycz MA et al. Regulation of eosinophil apoptosis by nitric oxide: Role of c-Jun-N-terminal kinase and signal transducer and activator of transcription 5. J Allergy Clin Immunol 2003; 112: 93–101. 12847485 10.1067/mai.2003.1587

[bib37] 37Pontin J, Blaylock MG, Walsh GM, Turner SW. Sputum eosinophil apoptotic rate is positively correlated to exhaled nitric oxide in children. Pediatr Pulmonol 2008; 43: 1130–1134. 18972415 10.1002/ppul.20921

[bib38] 38Ilmarinen-Salo P, Moilanen E, Kankaanranta H. Nitric oxide induces apoptosis in GM-CSF-treated eosinophils via caspase-6-dependent lamin and DNA fragmentation. Pulm Pharmacol Ther 2010; 23: 365–371. 20380887 10.1016/j.pupt.2010.04.001

[bib39] 39Ilmarinen-Salo P, Moilanen E, Kinnula VL, Kankaanranta H. Nitric oxide-induced eosinophil apoptosis is dependent on mitochondrial permeability transition (mPT), JNK and oxidative stress: apoptosis is preceded but not mediated by early mPT-dependent JNK activation. Respir Res 2012; 13: 73. 22920281 10.1186/1465-9921-13-73 PMC3495716

[bib40] 40Ilmarinen P, Moilanen E, Kankaanranta H. Mitochondria in the center of human eosinophil apoptosis and survival. Int J Mol Sci 2014; 15: 3952–3969. 24603536 10.3390/ijms15033952 PMC3975377

[bib41] 41Matera MG, Calzetta L, Cazzola M. TNF-alpha inhibitors in asthma and COPD: we must not throw the baby out with the bath water. Pulm Pharmacol Ther 2010; 23: 121–128. 19853667 10.1016/j.pupt.2009.10.007

[bib42] 42Yang M, Hogan SP, Mahalingam S, Pope SM, Zimmermann N, Fulkerson P et al. Eotaxin-2 and IL-5 cooperate in the lung to regulate IL-13 production and airway eosinophilia and hyperreactivity. J Allergy Clin Immunol 2003; 112: 935–943. 14610483 10.1016/j.jaci.2003.08.010

[bib43] 43Klein A, Talvani A, Silva PM, Martins MA, Wells TN, Proudfoot A et al. Stem cell factor-induced leukotriene B4 production cooperates with eotaxin to mediate the recruitment of eosinophils during allergic pleurisy in mice. J Immunol 2001; 167: 524–531. 11418691 10.4049/jimmunol.167.1.524

[bib44] 44Kobayashi T, Iijima K, Kita H. Marked airway eosinophilia prevents development of airway hyper-responsiveness during an allergic response in IL-5 transgenic mice. J Immunol 2003; 170: 5756–5763. 12759459 10.4049/jimmunol.170.11.5756

[bib45] 45Rosenberg HF, Phipps S, Foster PS. Eosinophil trafficking in allergy and asthma. J Allergy Clin Immunol 2007; 119: 1303–1310. 17481712 10.1016/j.jaci.2007.03.048

[bib46] 46Yamaguchi Y, Hayashi Y, Sugama Y, Miura Y, Kasahara T, Kitamura S et al. Highly purified murine interleukin 5 (IL-5) stimulates eosinophil function and prolongs *in vitro* survival. IL-5 as an eosinophil chemotactic factor. J Exp Med 1988; 167: 1737–1742. 2835420 10.1084/jem.167.5.1737 PMC2188945

[bib47] 47Kankaanranta H, Ilmarinen P, Zhang X, Adcock IM, Lahti A, Barnes PJ et al. Tumour necrosis factor-alpha regulates human eosinophil apoptosis via ligation of TNF-receptor 1 and balance between NF-kappaB and AP-1. PloS One 2014; 9: e90298. 24587316 10.1371/journal.pone.0090298 PMC3938678

[bib48] 48Huaux F, Gharaee-Kermani M, Liu T, Morel V, McGarry B, Ullenbruch M et al. Role of Eotaxin-1 (CCL11) and CC chemokine receptor 3 (CCR3) in bleomycin-induced lung injury and fibrosis. The American journal of pathology 2005; 167: 1485–1496. 16314464 10.1016/S0002-9440(10)61235-7 PMC1613185

[bib49] 49Sumi Y, Hamid Q. Airway remodeling in asthma. Allergol Int 2007; 56: 341–348. 17965577 10.2332/allergolint.R-07-153

[bib50] 50Foster PS, Mould AW, Yang M, Mackenzie J, Mattes J, Hogan SP et al. Elemental signals regulating eosinophil accumulation in the lung. Immunol Rev 2001; 179: 173–181. 11292021 10.1034/j.1600-065x.2001.790117.x

[bib51] 51Lora JM, Zhang DM, Liao SM, Burwell T, King AM, Barker PA et al. Tumor necrosis factor-alpha triggers mucus production in airway epithelium through an IkappaB kinase beta-dependent mechanism. J Biol Chem 2005; 280: 36510–36517. 16123045 10.1074/jbc.M507977200

[bib52] 52Pinho V, Souza DG, Barsante MM, Hamer FP, De Freitas MS, Rossi AG et al. Phosphoinositide-3 kinases critically regulate the recruitment and survival of eosinophils *in vivo*: importance for the resolution of allergic inflammation. J Leuk Biol 2005; 77: 800–810. 10.1189/jlb.0704386 15860799

[bib53] 53Perez DA, Vago JP, Athayde RM, Reis AC, Teixeira MM, Sousa LP et al. Switching off key signaling survival molecules to switch on the resolution of inflammation. Mediators Inflamm 2014; 2014: 829851. 25136148 10.1155/2014/829851 PMC4127222

[bib54] 54Pollock JD, Williams DA, Gifford MA, Li LL, Du X, Fisherman J et al. Mouse model of X-linked chronic granulomatous disease, an inherited defect in phagocyte superoxide production. Nat Genet 1995; 9: 202–209. 7719350 10.1038/ng0295-202

[bib55] 55Duffin R, Leitch AE, Sheldrake TA, Hallett JM, Meyer C, Fox S et al. The CDK inhibitor, R-roscovitine, promotes eosinophil apoptosis by down-regulation of Mcl-1. FEBS Lett 2009; 583: 2540–2546. 19616548 10.1016/j.febslet.2009.07.017

[bib56] 56Kurowska-Stolarska M, Kewin P, Murphy G, Russo RC, Stolarski B, Garcia CC et al. IL-33 induces antigen-specific IL-5+ T cells and promotes allergic-induced airway inflammation independent of IL-4. J Immunol 2008; 181: 4780–4790. 18802081 10.4049/jimmunol.181.7.4780

[bib57] 57Marques PE, Oliveira AG, Pereira RV, David BA, Gomides LF, Saraiva AM et al. Hepatic DNA deposition drives drug-induced liver injury and inflammation in mice. Hepatology 2014; 61: 348–360. 24824608 10.1002/hep.27216

[bib58] 58Serra MF, Anjos-Valotta EA, Olsen PC, Couto GC, Jurgilas PB, Cotias AC et al. Nebulized lidocaine prevents airway inflammation, peribronchial fibrosis, and mucus production in a murine model of asthma. Anesthesiology 2012; 117: 580–591. 22846675 10.1097/ALN.0b013e31826687d5

[bib59] 59Arantes-Costa FM, Lopes FD, Toledo AC, Magliarelli-Filho PA, Moriya HT, Carvalho-Oliveira R et al. Effects of residual oil fly ash (ROFA) in mice with chronic allergic pulmonary inflammation. Toxicol Pathol 2008; 36: 680–686. 18477768 10.1177/0192623308317427

[bib60] 60Sousa LP, Carmo AF, Rezende BM, Lopes F, Silva DM, Alessandri AL et al. Cyclic AMP enhances resolution of allergic pleurisy by promoting inflammatory cell apoptosis via inhibition of PI3K/Akt and NF-kappaB. Biochem Pharmacol 2009; 78: 396–405. 19422809 10.1016/j.bcp.2009.04.030

[bib61] 61Strath M, Warren DJ, Sanderson CJ. Detection of eosinophils using an eosinophil peroxidase assay. Its use as an assay for eosinophil differentiation factors. J Immunol Methods 1985; 83: 209–215. 3840509 10.1016/0022-1759(85)90242-x

[bib62] 62Olsen PC, Ferreira TP, Serra MF, Farias-Filho FA, Fonseca BP, Viola JP et al. Lidocaine-derivative JMF2-1 prevents ovalbumin-induced airway inflammation by regulating the function and survival of T cells. Clin Exp Allergy 2011; 41: 250–259. 20874831 10.1111/j.1365-2222.2010.03580.x

