# Supplementary figures and images for: Induction of eosinophil apoptosis by hydrogen peroxide promotes the resolution of allergic inflammation
Source: Cell Death Dis. 2015 Feb 12;6(2):e1632–. doi: 10.1038/cddis.2014.580 (PMC4669804; doi:10.1038/cddis.2014.580)

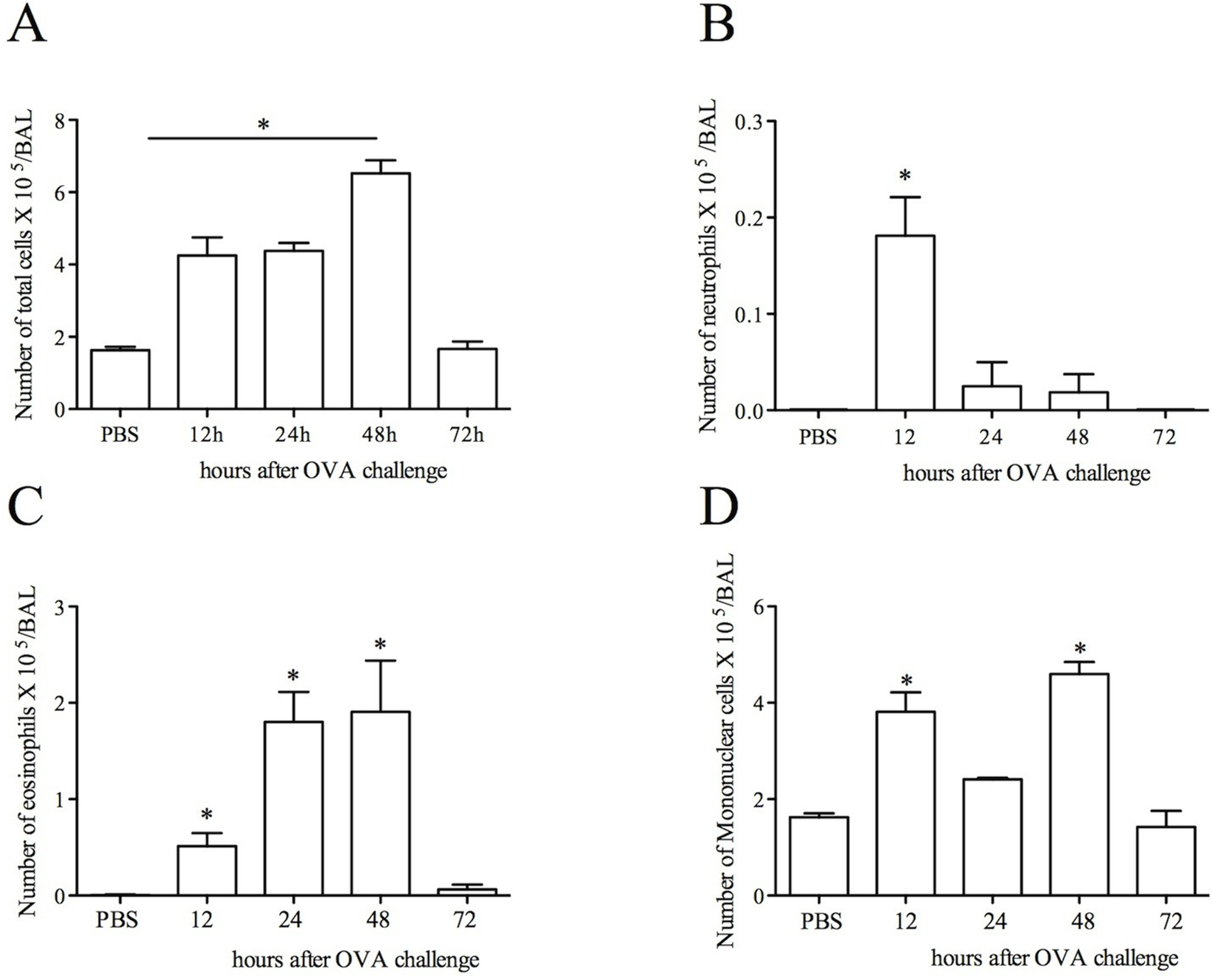

Supplement: Supplementary Figure 1 [file cddis2014580x1.tif]

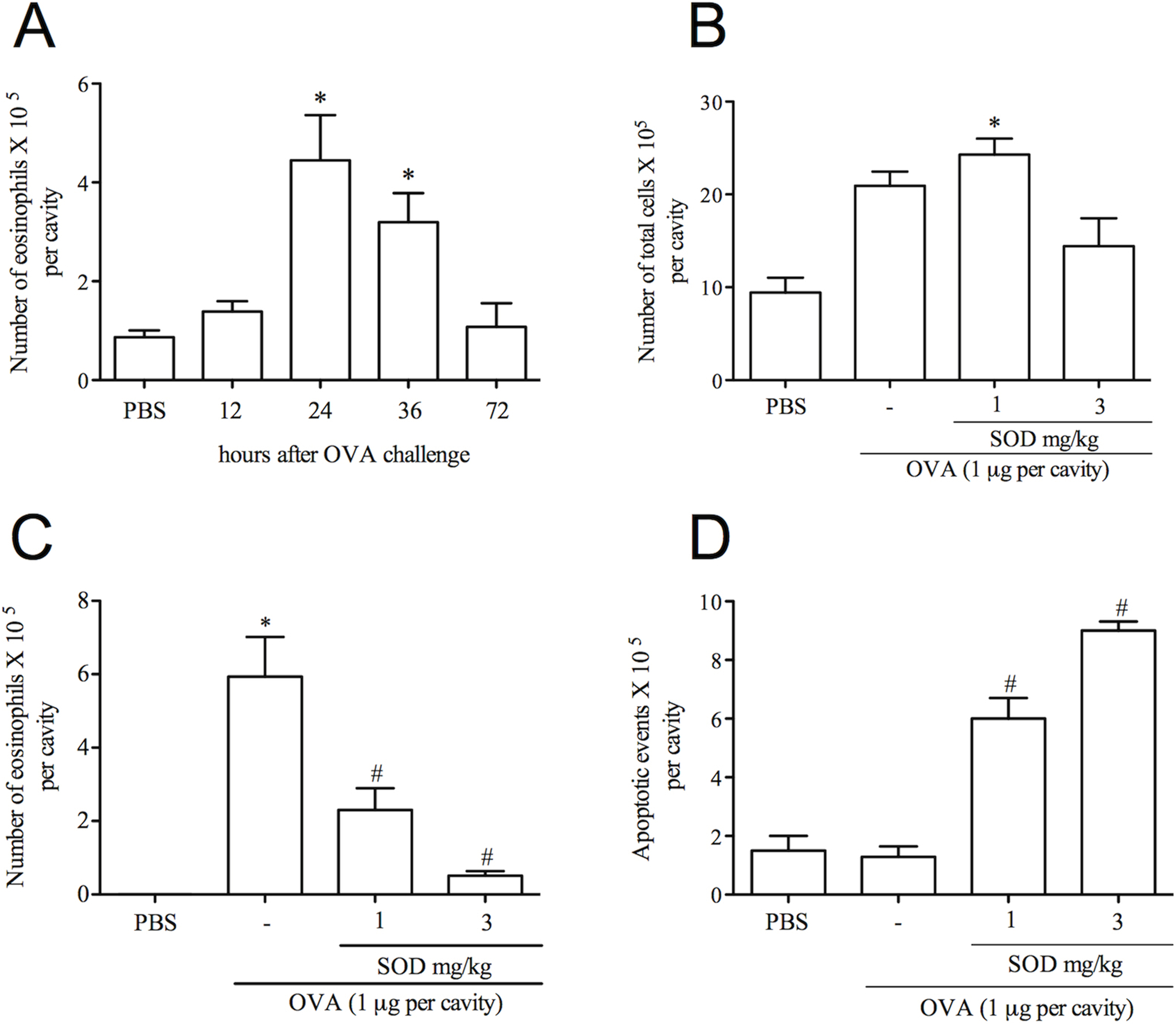

Supplement: Supplementary Figure 2 [file cddis2014580x2.tif]

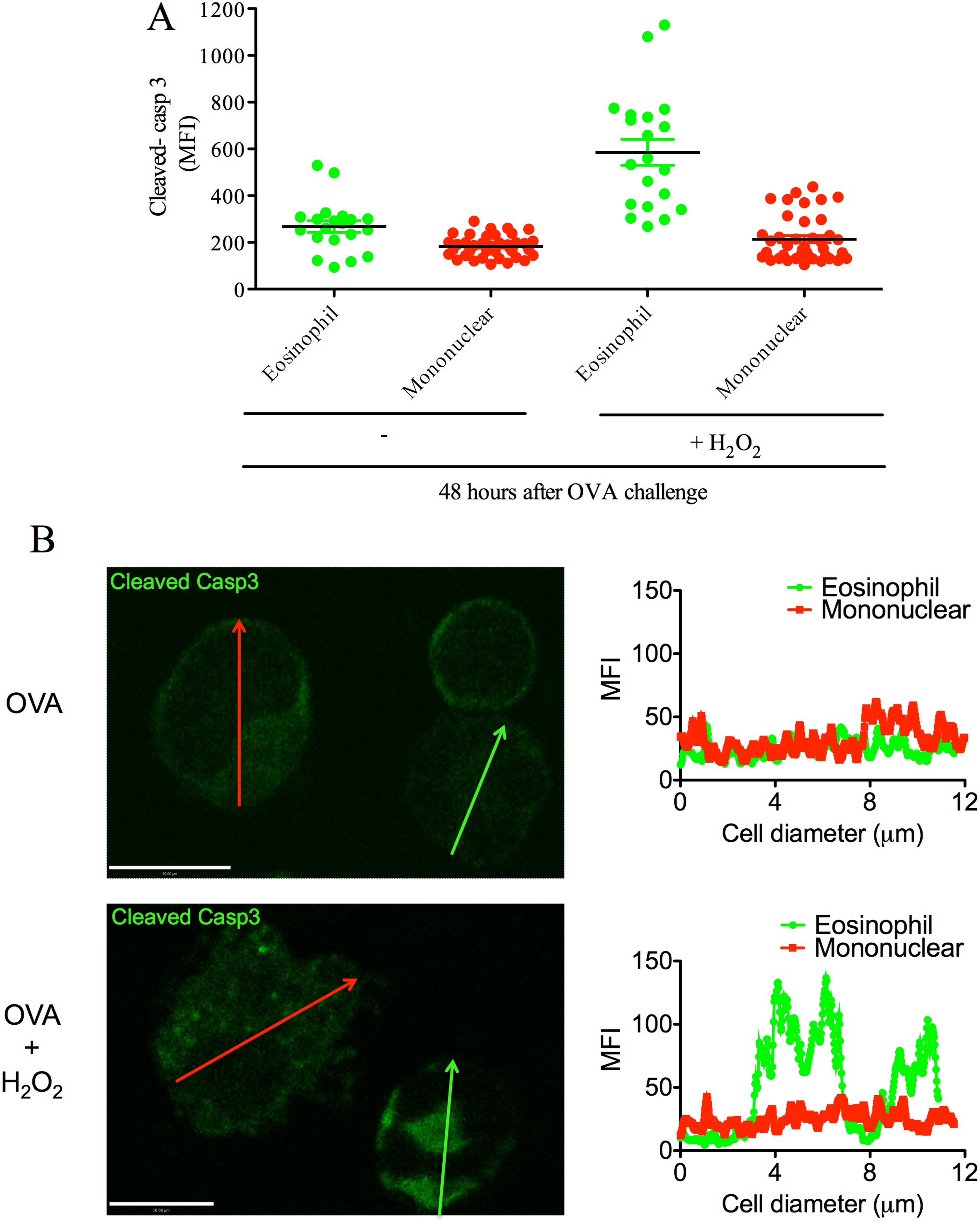

Supplement: Supplementary Figure 3 [file cddis2014580x3.tif]

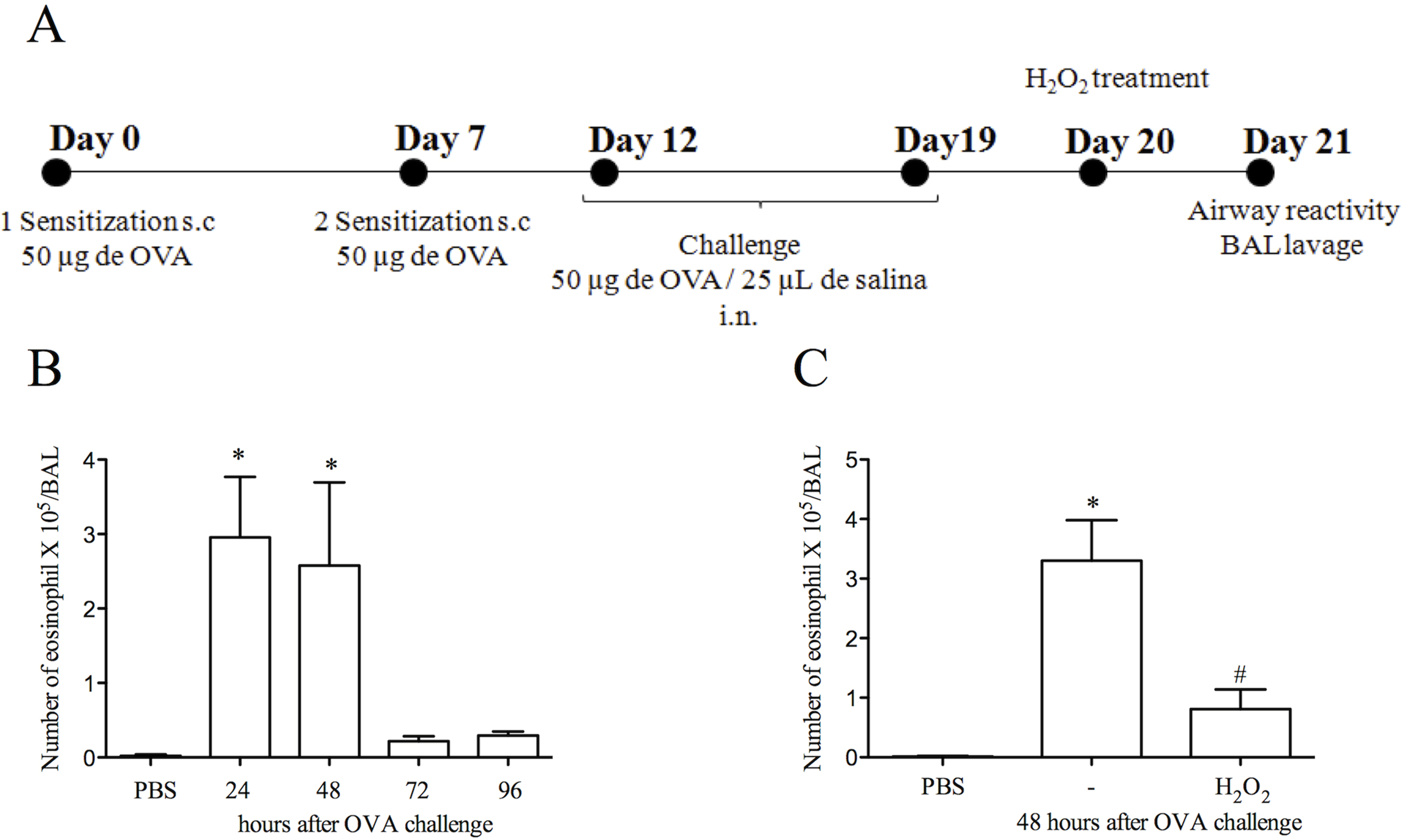

Supplement: Supplementary Figure 4 [file cddis2014580x4.tif]

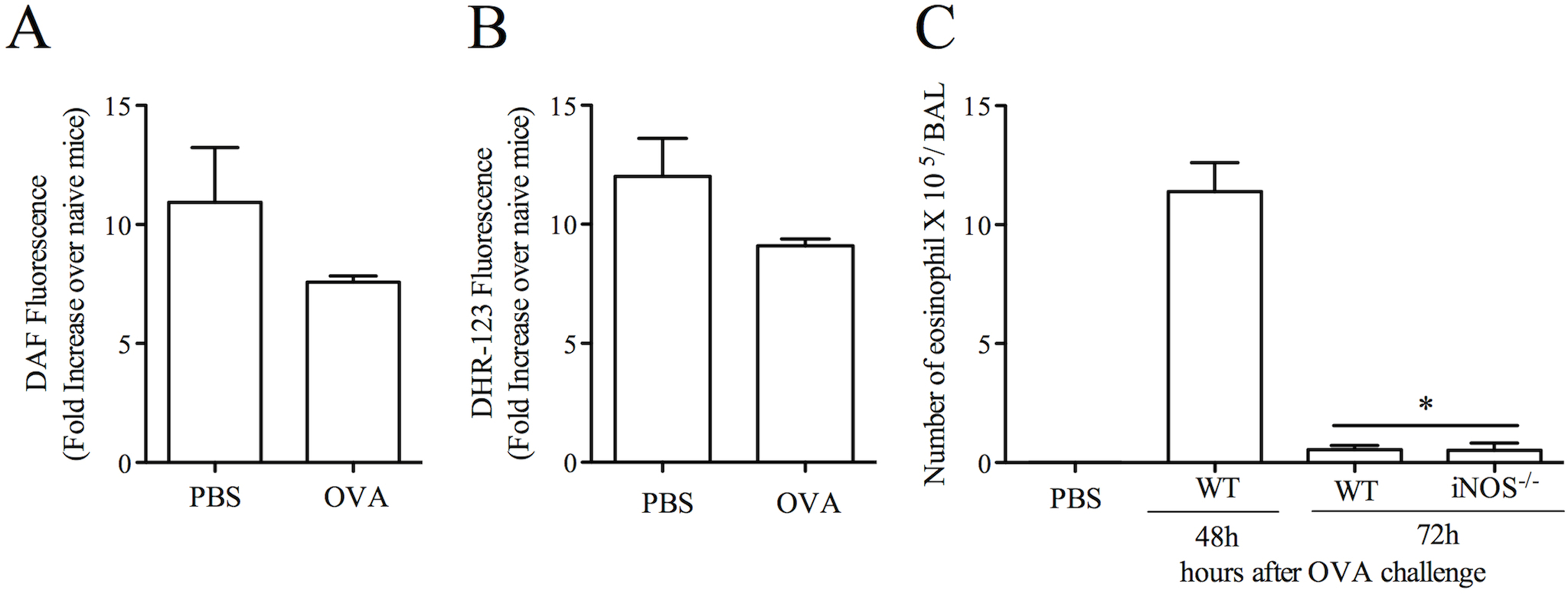

Supplement: Supplementary Figure 5 [file cddis2014580x5.tif]
