# Supplementary material for: Induction of eosinophil apoptosis by hydrogen peroxide promotes the resolution of allergic inflammation
Source: Cell Death Dis. 2015 Feb 12;6(2):e1632–. doi: 10.1038/cddis.2014.580 (PMC4669804; doi:10.1038/cddis.2014.580)
Supplement: Supplementary Figure Legends [file cddis2014580x6.pdf]

Supplemental Figure 1.

Characterization of the inflammatory response in a mouse model of asthma. The numbers of leukocytes in BAL were assessed in different time points in WT mice.

Number of total cells (A) neutrophils (B), eosinophils (C) and mononuclear (D) are depicted. Negative controls were mice challenged with sterile PBS. Data represent mean ±SEM (n=5) * = $P < 0.05$ versus control mice.

Supplemental Figure 2.

Kinetics of recruitment of inflammatory cells and apoptotic events in an allergic pleurisy model. (A). Kinetics of recruitment of eosinophils in pleural cavity (B). Treatment with superoxide dismutase (SOD; 3mg/kg or 1mg/kg*). Eosinophils (C) and apoptotic events (D). $P <0.05$ when compared to vehicle treated animals. Data represent mean ±SEM (n=5) * = $P < 0.05$ versus control mice.

Supplemental Figure 3.

H_2O_2 preferentially induces apoptosis in eosinophils. (A) Fluorescence quantification in BAL-derived leukocytes showing a significant increase in cleaved caspase 3 staining (a marker for apoptosis) in eosinophils in comparison to mononuclear cells due to H_2O_2 treatment (48hs after OVA challenge). (B) Fluorescence profile analysis of BAL leukocytes from OVA-challenged H_2O_2-treated mice revealed a spread, higher intracellular signal for cleaved caspase 3 in eosinophils in comparison to mononuclear cells. Arrows show the region whose fluorescence profile was assessed. 60x. Scale = 10□m.

Supplemental Figure 4.

Kinetics of recruitment of inflammatory cells and apoptotic events in a mouse model of asthma with eight OVA challenges. Protocol of asthma induction and treatment (A). Kinetics of recruitment of eosinophils in BAL (B). Number of eosinophils after

H_2O_2 treatment (0.5M) . P <0.05 when compared to vehicle treated animals. Data represent mean ±SEM (n=5) * = P < 0.05 versus control mice.

Supplemental Figure 5.

Production of reactive nitrogen species and kinetics of eosinophils in asthma model. Fluorescence intensity of DAF (A) DHR-123 (B) 48 h after OVA challenge. Eosinophils 48 and 72 h after induction of asthma in WT mice (-), and 72 h in iNOS-/ mice (C). Data represent mean ±SEM (n=5) * = P < 0.05 versus control mice.
